# Genome-wide analysis and functional characterization of *AsTPS* genes in Chinese angelica (*Angelica sinensis*)

**DOI:** 10.3389/fpls.2026.1872863

**Published:** 2026-07-10

**Authors:** Heng Xiang, Mengqi Sui, Anqi Wang, Junzhuo Zhang, Shengli Yao, Chao Xiong, Ran Xu

**Affiliations:** School of Life Science and Technology, Wuhan Polytechnic University, Wuhan, China

**Keywords:** *Angelica sinensis*, functional characterization, genome-wide analysis, terpene synthase, volatile terpenoids

## Abstract

*Angelica sinensis* (*Oliv*.) Diels is a medicinal plant rich in volatile terpenoids with tissue-specific distribution. Terpene synthase (TPS) is recognized as a major enzyme in terpenoid biosynthesis. However, the correspondence between the *AsTPS* copy and specific terpenoids in *A. sinensis* remains poorly understood. Here, we annotated 27 *AsTPS* genes in the *A. sinensis* genome, several of which showed strong co-expression with terpenoid accumulation. Four *AsTPS* genes were selected for functional validation. Full-length or N-terminally truncated open reading frames were heterologously expressed in *E. coli*, and *in vitro* enzymatic assays coupled with gas chromatography–mass spectrometry confirmed that *AsTPS*10 and *AsTPS*12 catalyze *β-myrcene* and (E)-*β-farnesene* synthesis from geranyl diphosphate and farnesyl diphosphate, respectively. Our findings highlight candidate genes involved in volatile terpenoid compound biosynthesis, providing a basis for the development and application of monoterpenes and sesquiterpenes in *A. sinensis*.

## 1 Introduction

*Angelica sinensis* (*Oliv.*) Diels (2n=2x=22) is a medicinal plant of the *Apiaceae* family (Xu et al., 2020b). It has been widely used in Chinese traditional medicine, first described in *The Divine Farmer’s Classic of Materia Medica* (Nugent-Head, 2014). Dried roots of *A. sinensis*, referred to as “Dang Gui” and “female ginseng” in China, have served as remedies for blood and digestive disorders (Manjir and Kakoti, 2015). Owing to its anti-inflammatory and antioxidant properties (Hook, 2014; Wei et al., 2016; Li et al., 2022a), it is extensively used in modern food and cosmetics for its roles in improving immune function and facilitating skin metabolism.

More than 140 chemicals, including both volatile organic compounds and non-volatile compounds (Wei et al., 2016), have been identified in the dried roots of *A. sinensis*. Among them, VOCs, mainly composed of monoterpenes and sesquiterpenes, are regarded as the major bioactive and characteristic components in *A. sinensis*. These two types of volatile terpenoids possess diverse bioactivity, including antioxidant, anti-tumor, immunomodulatory, and antidiabetic effects (Wei et al., 2016; Li et al., 2022c). Although there have been significant advances in extraction, chemical structure, and pharmacological effects of terpenoids, the biosynthetic pathways and related genes for the biosynthesis of these two types of volatile terpenoids require further characterization.

In plants, volatile terpenoid precursors are all derived from two separate pathways: the mevalonate pathway (MVA) in the cytoplasm and the plastidial methylerythritol phosphate pathway (MEP) (Chen et al., 2011; Yan et al., 2023). Terpene synthases (TPSs) catalyze the final conversion of geranyl diphosphate (GPP), farnesyl diphosphate (FPP), and all-trans-geranylgeranyl diphosphate (GGPP) into various sesquiterpenes, monoterpenes, diterpenes, and their derivatives, making them critical enzymes in both pathways (Xu et al., 2017b; Liu et al., 2025).

Studies across various terrestrial plants have identified plant *TPSs* encoding a distinct gene family that participates in terpenoid biosynthesis (Yu et al., 2020; Xu et al., 2024). Seven subfamilies make up the *TPS* gene family: *TPS*-a, *TPS*-b, *TPS*-c, *TPS*-d, *TPS*-e/f, *TPS*-g, and *TPS*-h (Trapp and Croteau, 2001; Diao et al., 2022). These subfamilies are distributed in gymnosperms, angiosperms, and certain vascular plants (Vandesteene et al., 2010; Chen et al., 2011; Tholl and Lee, 2011). *TPS* genes occur in *Arabidopsis thaliana*, *Vitis vinifera*, tomato, rice, and so on (Kaundal et al., 2010; Martin et al., 2010; Falara et al., 2011; Xu et al., 2017b; Zhang et al., 2017; Liu et al., 2024; Zhou et al., 2025). Previous studies have shown that among the subfamilies, the *TPS-a* genes are the most abundant. For example, in *A. thaliana and Solanum lycopersicum*, the largest subfamily of *TPS* genes is *TPS*-a (Falara et al., 2011). The *TPS-*b subfamily is mainly composed of angiosperm monoterpene synthase genes (Dudareva et al., 2013). *TPS-*d and *TPS-*h are distributed only in *Gymnosperms* and *Selaginella moellend*, respectively (Chen et al., 2011). Furthermore, *TPS* gene family data is becoming increasingly available for several plant species through individual genome screening (Chen et al., 2011; Falara et al., 2011; Liu et al., 2024; Xu et al., 2024; Liu et al., 2025; Zhou et al., 2025), yet only a few *AsTPS* genes have been found in *Angelica*, a genus within the *Apiaceae* family.

Here, a comprehensive analysis of *A. sinensis* root VOCs and transcriptomes identified essential genes in the MVA and MEP pathways. Twenty-seven *AsTPS* genes were annotated in the *A. sinensis* genome (Han et al., 2022; Li et al., 2023). Among them, four key candidate genes were identified through the association of gene expression and chemical quantification. The functions of two of these genes in the synthesis process of monoterpenes and sesquiterpenes were confirmed through the prokaryotic expression experiment. This study offers novel insights into the genetic mechanisms underlying terpenoid biosynthesis in *A. sinensis*.

## 2 Materials and methods

### 2.1 Plant materials and reagents

Two-year-old *A. sinensis* plants were obtained in August from Enshi (109°55′E, 30°07′N; altitude: 1800~2000 m; average temperature:15.5°C; average annual precipitation: 1400~1600 mm), Hubei Province, China. Associate professor Ran Xu (School of Life Science and Technology, Wuhan Polytechnic University, China) positively identified these samples as *A. sinensis*. The plant roots were washed and then separated into three tissues (periderm, cortex, and stele) prior to downstream analysis, with three replicates for each tissue type. Each sample was then manually separated into two parts. One part was immediately frozen in liquid nitrogen and stored at −20°C for volatile terpenoid metabolomic investigations, and the other was stored at −80°C for transcriptomic analyses until use.

### 2.2 Determination of metabolites

The tissue samples were ground using liquid nitrogen (periderm, cortex, and stele), then vortexed to guarantee uniform mixing, and finally approximately 500 mg of each sample was transferred into a headspace vial. The temperature was kept steady at 60 °C, and the samples were shaken for 5 min, followed by placing the extraction head (DVB/CWR/PDMS) into the headspace bottle, and carrying out headspace extraction for 15 min. At 250 °C, the samples were analyzed for 5 min, which was followed by GC-MS separation and identification (DB-5MS, 30 m × 0.25 mm × 0.25 μm, Agilent J&W Scientific, Folsom, CA, USA). High-purity helium was used at a uniform flow rate of 1.2 mL/min. The heating procedure was carried out as: heating at a temperature of 40 °C for 3.5 min, increasing the temperature to 100 °C at 10 °C/min, then increasing it to 180 °C at 7 °C/min, and finally to 280 °C at 25 °C/min, and maintaining it at 280 °C for 5 min. The mass spectrometer was set to full-scan (SCAN) mode at m/z 50–500 amu, and the electron energy was adjusted to 70 eV. Using mass spectrometry, qualitative and semi-quantitative studies of metabolites in the sample were performed, based on the NIST database.

### 2.3 Differential metabolites selected

Significantly regulated metabolites between groups were determined by VIP≥1 and absolute Log2FC (fold change)≥1. VIP values were extracted from the OPLS-DA result, which also contains score plots and permutation plots, was generated using the R package MetaboAnalystR. The data were log transformed (log2) and mean centered before OPLS-DA. In order to avoid overfitting, a permutation test (200 permutations) was performed.

### 2.4 RNA isolation, library construction, and sequencing

The transcriptomic analysis *A. sinensis* root samples (periderm, cortex, and stele), with total RNA extracted from samples using Trizol. The approach used was based on methods described by Rio et al (Rio et al., 2010). The raw data, obtained via Illumina sequencing and CASAVA base-calling, were processed using Skewer and FastQC to yield clean reads. When the original transcriptome analysis was conducted, the current reference genome annotation of *A. sinensis* had not yet been incorporated into our analytical workflow. Therefore, Trinity (Grabherr et al., 2011) was used to perform *de novo* assembly of the reads from each sample under default settings. Gene expression quantification and DEG detection (FC≥2, FDR ≤ 0.05) were based on FPKM calculation.

### 2.5 Identification of genes involved in MVA and MEP pathway

We selected proteins from *A. thaliana* monoterpene and sesquiterpene biosynthesis pathways to identify MVA and MEP-related genes in the *A. sinensis* genome, and used BLASTP v2.10.0 (E-value<1e^-5^) to find orthologs. The fragments per kilobase of exon model per million mapped reads (FPKM) were calculated to quantify the expression level among the three tissues of *A. sinensis*. The false discovery rate (FDR) was used to compute the differences in the significance of transcript abundance. log2 ratio≥2 and FDR ≤ 0.05 were considered to identify differentially expressed genes (DEGs) (Differential expression analysis were carried out at the gene level). Heatmaps were constructed using R programming language and software (R 3.4.2, R packages).

### 2.6 *AsTPS* gene identification

The protein sequences of *A. sinensis* were obtained from previous genome data to build a local BLAST database (Han et al., 2022; Li et al., 2023). BLASTP alignment (*E*-value<10^-5^) of TPS protein sequences from rice (Sun et al., 2022), celery (Li et al., 2022b), and carrot (Muchlinski et al., 2020), and tomato (Falara et al., 2011) against the *A. sinensis* protein sequences was performed to identify candidate *AsTPS* genes. The Pfam results were combined with the local BLASTP search results, redundancies were removed from the obtained candidate gene sequences, and the sequences were imported into Apollo software for manual curation.

Candidate *AsTPS* sequences were further filtered according to complete open reading frames, clear start and stop codons, no premature stop codons or obvious frameshifts, and those with recognizable *AsTPS* conserved domains were retained as final *AsTPS* genes. The *AsTPS* N-terminal domain PF01397 and *TPS* C-terminal catalytic/metal-binding domain PF03936 were examined using HMMER/Pfam and CDD searches. Conserved *TPS* motifs, including DDxxD and NSE/DTE, were inspected through multiple sequence alignment, and the RR(x)_8_W motif was also examined, particularly for *AsTPS*-b and *AsTPS*-g members, although this motif is not universally conserved across all *TPS* subfamilies. Sequences with disrupted ORFs, severe ORF-disrupting truncations, premature stop codons, obvious frameshifts, or insufficient *TPS*-domain support were excluded. Several relatively short candidates were retained only when they showed intact ORFs, recognizable *TPS*-domain evidence, conserved motif support, and phylogenetic consistency with TPS proteins. The subcellular localization of the predicted *As*TPS proteins was predicted using the WoLF PSORT website (https://wolfpsort.hgc.jp/). The CDS sequences, gene IDs, and protein sequences for all candidate *AsTPS* genes are provided in the **Supplementary Table S14**.

Annotated TPS protein sequences from *A. sinensis* and four plant species were aligned ClustalW. Phylogenetic tree was constructed following Yang et al (Yang et al., 2023), and further refined using Evolview. We used the GTF/GFF Gene Location Visualizer plugin in TBtools to map the chromosomal locations of *A. sinensis* genes. Conserved domains in *A. sinensis* were predicted using the MEME online tool, and the results were visualized with TBtools. From the GFF file information of *A. sinensis* in the NCBI database, the TBtools software was used to visualize the exons/introns of the *AsTPS* gene.

### 2.7 Characterization of the *AsTPS* genes

Spearman correlation analysis (Langfelder and Horvath, 2008) was performed in Python to investigate the co-expression patterns: between sesquiterpenes and *TPS*-a, and between monoterpenes and *TPS*-b/*TPS*-g. A multiple testing correction step was included in the analysis, applying the false discovery rate (FDR) correction to the original *p*-values. Genes with |R|>0.80, *p* < 0.05, and high expression levels (FPKM>10) were selected for functional validation. The coding sequence of *AsTPS*10 and the N-terminally truncated coding sequences of *AsTPS*12, *AsTPS*16, and *AsTPS*27 were amplified for heterologous expression. According to the prediction results and primer design, the first 0, 30, 30, and 30 amino acids were removed from *AsTPS*10, *AsTPS*12, *AsTPS*16, and *AsTPS*27, respectively. The signal peptide-free ORFs of *AsTPS*10, *AsTPS*12, *AsTPS*16, and *AsTPS*27 were amplified using Taq DNA polymerase (2×Phanta Flash Master Mix, Vazyme). Primer information is shown in **Table 1**.

**Table 1:** pET14b-*AsTPSs* primer list.

Genes	Primers (5’ to 3’)
pET14b*-AsTPS10*-F	TGTACTTCCAGGGTCATATGATGGCTCTCCAAGGTTTGTTTTCAAC
pET14b*-AsTPS10*-R	CTTTGTTAGCAGCCGGATCCTCACTTGGTAAGAGTAAAGGGTTCCACTAAC
pET14b*-AsTPS12*-F	TGTACTTCCAGGGTCATATGATGTATCATCCCAGTGTTTGGGGAG
pET14b*-AsTPS12*-R	CTTTGTTAGCAGCCGGATCCTCATATCGGAACGGGATCAACCAG
pET14b*-AsTPS16*-F	TGTACTTCCAGGGTCATATGATGTGGGGAGACAAATTCCTCG
pET14b*-AsTPS16*-R	CTTTGTTAGCAGCCGGATCCTTACATATGGGGAATAGGATCTAACAGTATCAAGG
pET14b*-AsTPS27*-F	TGTACTTCCAGGGTCATATGATGTTCCAAGGTCTGTTTTGCCC
pET14b*-AsTPS27*-R	CTTTGTTAGCAGCCGGATCCCTAAAGATTGAAGGGTTCCAGCAACA

The products were subcloned into the BamHI/XhoI sites of the pET14b vector using ClonExpress II (Vazyme). *E. coli* Rosetta cells were transformed with recombinant plasmids and empty vector (control), and protein expression was induced overnight at 16 °C with 0.5 mM IPTG. Proteins were harvested and purified via Ni-NTA affinity chromatography, then validated by Coomassie staining and quantified using SDS-PAGE densitometry. Then, the protein concentration was determined using the NanoDrop instrument, and the protein concentration for the experiment was adjusted to be between 20–40 ug/ml. Enzyme activity was assayed following Shang et al (Shang et al., 2020). with minor modification, and 100 µL of chromatographic-grade n-hexane was used to extract the product for GC-MS analysis. Systematic experiments were conducted with control groups lacking substrate or enzyme, and main products were recorded. The substrates GPP and FPP used in the experiment were purchased from Sigma Company. The standard substance *β*-*myrcene* was obtained from Shanghai Yuan Ye Biotechnology Co., Ltd., while (E)-*β*-*farnesene* was purchased from Tianjin Xishen Biochemical Technology Co., Ltd.

## 3 Results

### 3.1 Metabolism analysis of volatile terpenoid contents in *A. sinensis*

**Figure 1:**
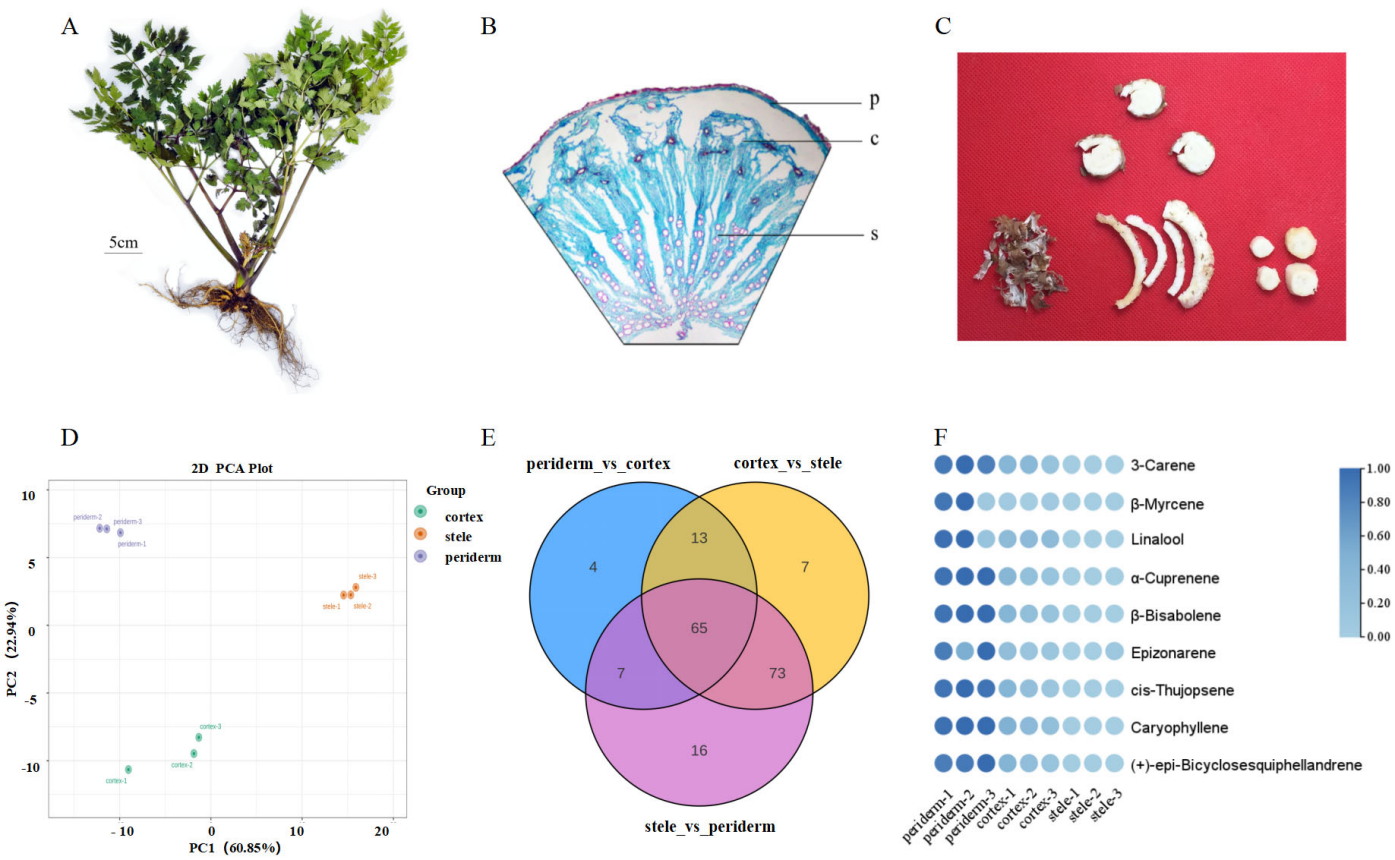
Analysis of VOCs in different tissues of *A. sinensis* (p: periderm, c: cortex, s: stele). **(A)** *A. sinensis* plants, **(B)** The cross-section of the root of *A. sinensis*. **(C)** Different root tissues of *A. sinensis*. **(D)** PCA score plot of metabolites detected by HS-SPME-GC-MS from different tissues. **(E)** Venn diagram showing number of metabolites that are significantly differentially accumulated between root tissues of *A. sinensis*. **(F)** Heat map of 3 terpenoid monoterpenes and 6 sesquiterpenoids in different tissues of *A. sinensis* (VIP>1, *p* < 0.05).

To explore the spatial distribution of the volatile compounds, we firstly quantified chemical contents of root tissues (periderm, cortex, and stele) (**Figures 1A–C**). Various VOCs, including alcohols, terpenes, aldehydes, esters, hydrocarbons, and acids, were identified (**Supplementary Figure S1**). In PCA, the first two principal components accounted for 60.85% and 22.94% (**Figure 1D**).

Among the 239 identified compounds, 185 (77.4%) were shared across the three tissues (**Supplementary Table S1**; **Supplementary Figure S2**). Meanwhile, 65 metabolites were significantly differentially accumulated across the three root tissues (**Figure 1E**).

VOCs were detected in all tissues and the periderm exhibited the highest volatile compound content (**Supplementary Table S2**). A total of 41 terpenoids were identified in *A. sinensis* roots, including 10 monoterpenes and 31 sesquiterpenes (**Supplementary Table S3**). Among the three different root tissues, we identified 21 terpenoids, including 6 monoterpenes and 15 sesquiterpenes, that exhibited significant differential accumulation across the periderm, cortex, and stele (VIP>1, *p* < 0.05; **Supplementary Tables S4–S6**). Furthermore, 3 monoterpenoids and 6 sesquiterpenoids displayed the highest accumulation levels in the periderm, suggesting potential tissue - specific metabolic regulation (**Figure 1F**; **Supplementary Tables S4–S6**).

### 3.2 Tissue-specific gene expression patterns in *A. sinensis*

**Figure 2:**
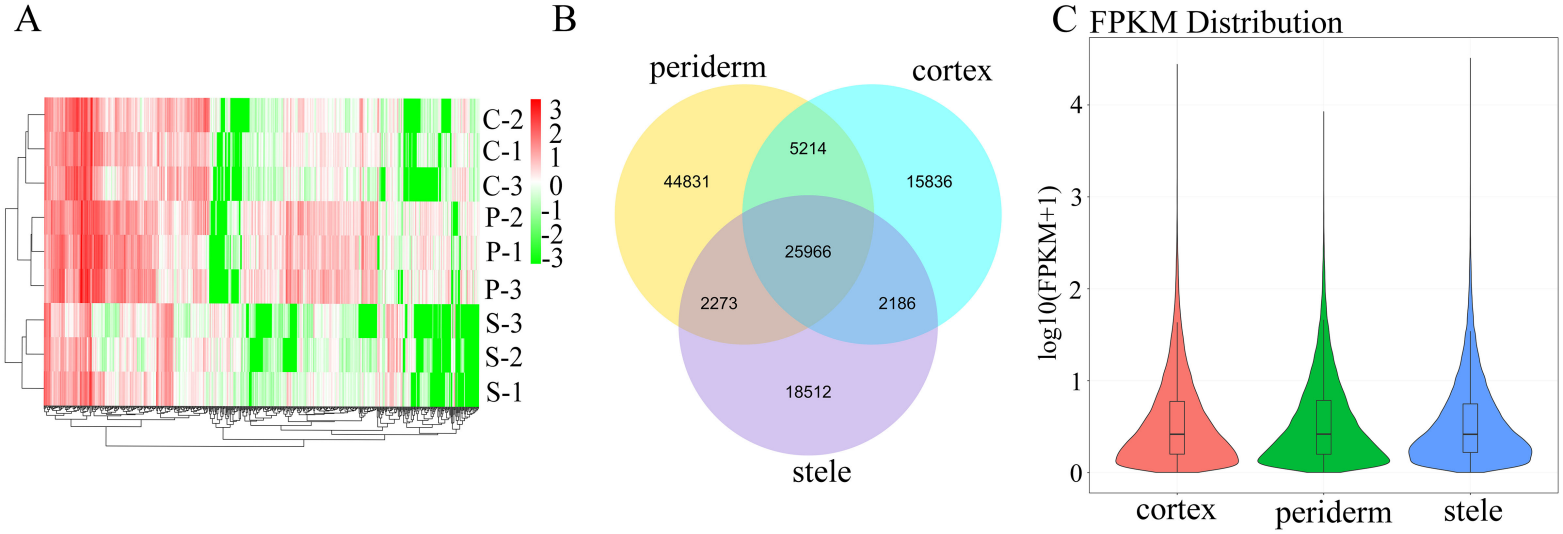
Cluster heatmap showing gene expression patterns across three tissue samples (p, periderm; c, cortex; s, stele) from *A. sinensis*. **(A)** Gene expression clustering tree in three *A. sinensis* tissues. **(B)** A Venn diagram comparing gene expression in different *A. sinensis* tissues. **(C)** Distribution of gene numbers across FPKM intervals in three different tissue samples.

Using individual transcript FPKM values, we further assessed the differential gene expression patterns across three *A. sinensis* tissue types with the aid of cluster heatmaps and Venn diagrams. The heatmap indicated that the periderm, cortex, and stele samples represented distinct groups (**Figure 2A**).

In the Venn diagram, the three root tissue types expressed 25,966 out of 114,845 transcripts, while 44,831, 15,863, and 18,512 were uniquely expressed in periderm, cortex, and stele samples, respectively. This suggests distinct differential gene expression patterns in *A. sinensis* root tissues (**Figure 2B**).

The number of genes and their proportions within different FPKM intervals for each sample were counted. The proportions of highly expressed genes with FPKM>1 in periderm, cortex, and stele samples were 17.63%, 17.61%, and 12.83%, respectively. These genes were used to identify the key genes of the MEP and MVA pathways in different tissues (**Figure 2C**).

### 3.3 Identification of transcripts involved in volatile terpenoids biosynthesis in *A. sinensis* roots

A total of 74 transcripts in the MEP and MVA pathways were assembled by homologous search. In the MEP pathway, 54.5% of the genes exhibited the highest expression level in the periderm, and cortical expression was relatively higher in another 25.5% of the genes. In the MVA pathway, 55.6% of the genes had the highest expression level in the periderm, while 22.2% had higher expression levels in the cortex.

**Figure 3:**
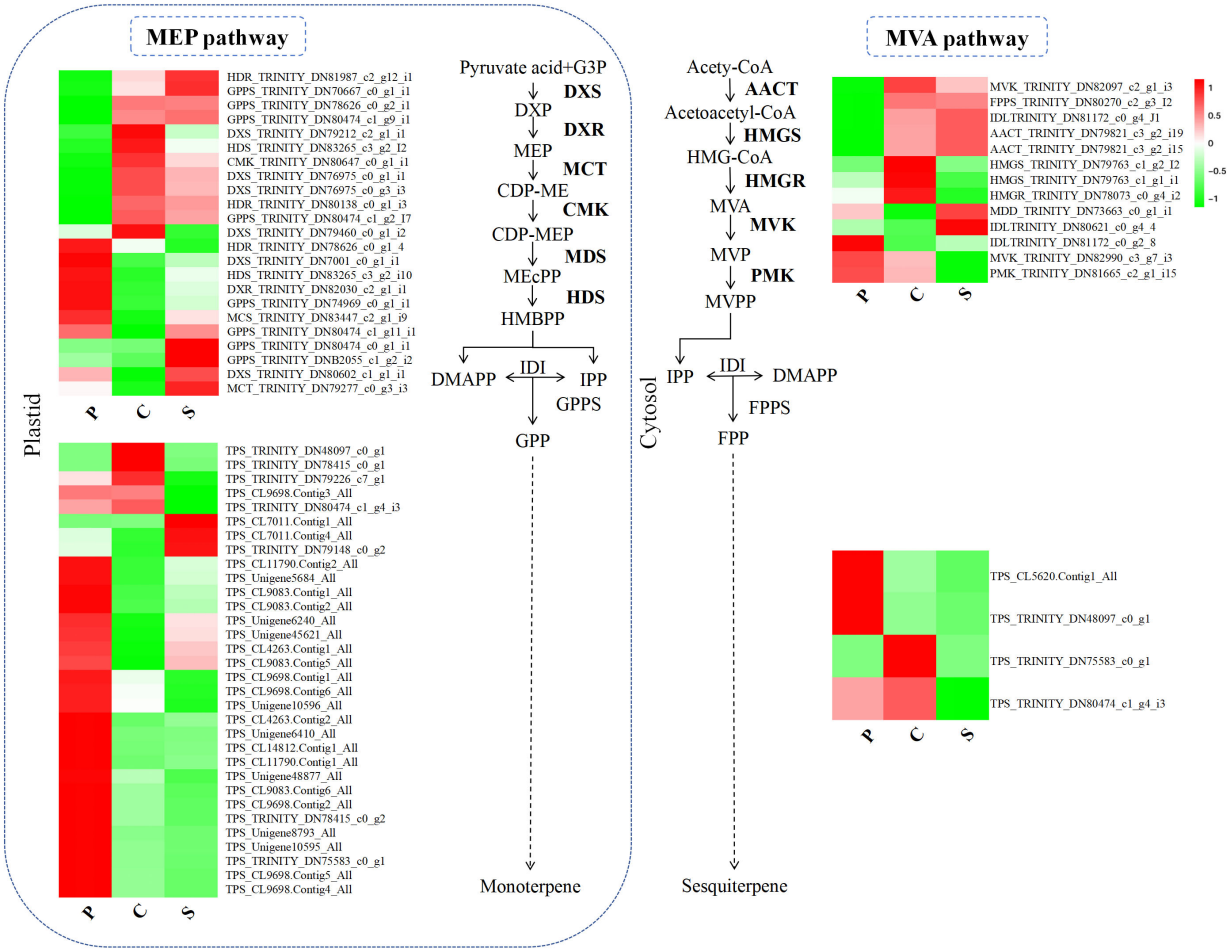
Expression heatmap of the assembled terpenoid biosynthetic transcripts following the MVA and MEP pathways.

It is noteworthy that most downstream genes exhibit significant tissue specificity (**Figure 3**; **Supplementary Tables S7, S8**) (FPKM≥1). The divergent expression patterns of these genes imply that the biosynthesis and accumulation of terpenoid compounds in *A. sinensis* roots are tissue-specific, dictating distinct metabolic profiles and different therapeutic potentials of these root tissues.

### 3.4 Genome-wide identification of *AsTPS* genes

To facilitate gene cloning and functional analysis, we retrieved 47 annotated terpene synthase (*TPS*) genes from the published *A. sinensis* genome. These genes were imported into the Apollo software and manually curated based on homolog information from *A. graveliens* and *C. sativum*. The resultant proteins were then submitted to the Conserved Domain Database (CDD) to identify conserved domains. After removing redundant, incomplete, severely truncated, and putative pseudogenized *TPS*-like sequences, a total of 27 *AsTPS*s with intact open reading frames were identified and renamed in ascending order of their chromosomal locations. (**Table 2**).

**Table 2:** Characteristics of *AsTPS* family members in *A. sinensis*.

Gene name	No. of amino acids (aa)	Isoelectric point (pI)	Molecular mass/kDa	hydrophilicity	Predicted subcellular localization	*AsTPS* family
*AsTPS1*	487	6.49	56.283	-0.060	Extracellular fluid	*TPS*-a
*AsTPS2*	551	5.52	64.17	-0.426	Cytoplasm	*TPS*-b
*AsTPS3*	204	5.02	24.092	-0.481	Cytoplasm	*TPS*-b
*AsTPS4*	244	5.57	28.317	-0.407	Chloroplast	*TPS*-a
*AsTPS5*	220	8.69	26.319	-0.092	Cytoplasm	*TPS*-a
*AsTPS6*	562	5.62	64.641	-0.265	Cytoplasm	*TPS*-b
*AsTPS7*	564	5.35	64.913	-0.293	Nucleus	*TPS-*b
*AsTPS8*	644	5.87	74.271	-0.282	Endoplasmic reticulum	*TPS*-e/f
*AsTPS9*	786	5.85	89.815	-0.278	Nucleus	*TPS*-e/f
*AsTPS10*	591	5.64	67.852	-0.36	Plasmalemma	*TPS*-b
*AsTPS11*	586	5.77	67.852	-0.386	Mitochondrion	*TPS*-b
*AsTPS12*	569	5.17	65.793	-0.193	Chloroplast	*TPS*-a
*AsTPS13*	566	5.40	64.938	-0.139	Extracellular fluid	*TPS*-a
*AsTPS14*	430	5.48	49.690	-0.178	Nucleus	*TPS-*b
*AsTPS15*	535	6.58	61.727	-0.318	Cytoplasm	*TPS*-e/f
*AsTPS16*	566	5.88	65.747	-0.269	Cytoplasm	*TPS*-a
*AsTPS17*	560	5.90	65.482	-0.372	Chloroplast	*TPS*-b
*AsTPS18*	319	6.81	36.980	-0.216	Cytoplasm	*TPS*-g
*AsTPS19*	287	5.74	33.118	-0.313	Mitochondrion	*TPS*-b
*AsTPS20*	554	5.49	65.213	-0.283	Cytoplasm	*TPS*-b
*AsTPS21*	588	5.28	68.538	-0.293	Cytoplasm	*TPS*-b
*AsTPS22*	291	4.63	34.610	-0.336	Chloroplast	*TPS-*b
*AsTPS23*	497	4.93	57.898	-0.221	Nucleus	*TPS*-b
*AsTPS24*	718	5.64	83.028	-0.321	Peroxisome	*TPS*-c
*AsTPS25*	503	5.60	57.914	-0.313	Cytoplasm	*TPS*-b
*AsTPS26*	564	5.58	65.324	-0.232	Chloroplast	*TPS*-a
*AsTPS27*	618	5.57	71.383	-0.306	Chloroplast	*TPS*-b

**Figure 4:**
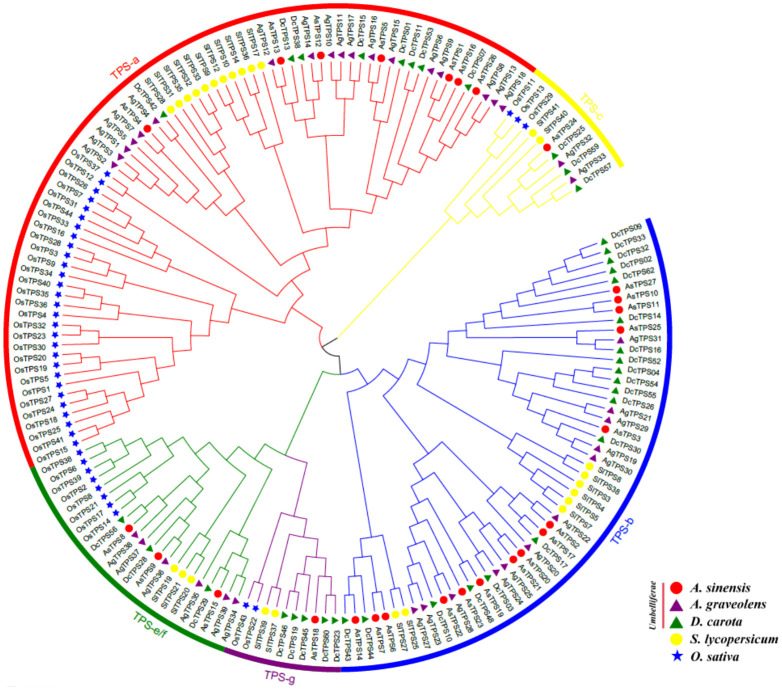
Phylogenetic tree of *TPS* genes across five plant species. The tree was constructed using annotated TPS protein sequences from *A. sinensis*, celery (*A. graveolens)*, carrot (*D. carota*), tomato (*S. lycopersicum*), and rice (*O. sativa*).The color-coded outer ring corresponds to distinct TPS functional classes: red for TPS-a, blue for TPS-b, yellow for TPS-c, purple for TPS-g and green for TPS-e/f.

In total five subfamilies were covered by these *AsTPS*, including *TPS*-a, *TPS*-b, *TPS*-g, *TPS*-c, *TPS*-e/f, which participated in biosynthesis of sesquiterpenoids, monoterpenes, and diterpenes, respectively (**Figure 4**).

**Figure 5:**
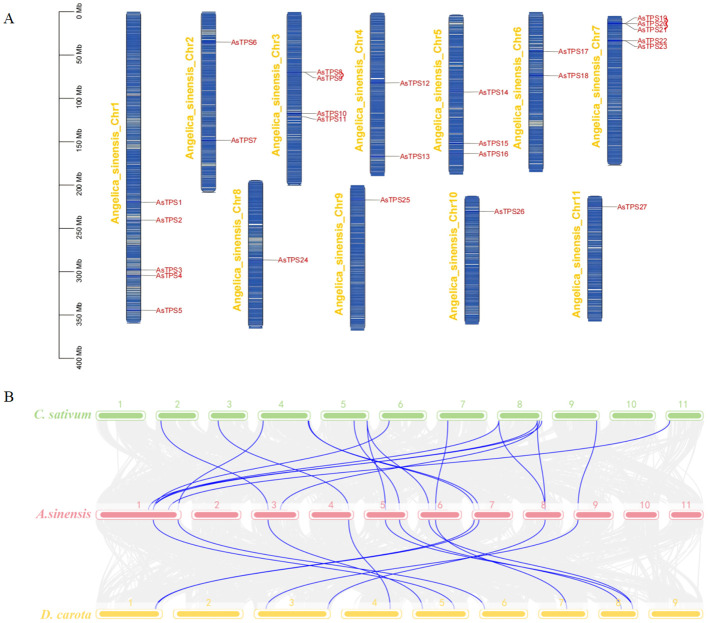
Chromosome localization of the 27 *AsTPS* gene family and analysis of gene homology with related species of the *Apiaceae* family. **(A)** Chromosomal localization and duplication events of *AsTPS* gene. (The gray areas mean good homology). **(B)** The gene homology map of *C. sa*tivum, *A. sinensis*, and *D. carota* (The gray areas mean good homology, and the blue line represents the *AsTPS* gene pairs).

It turned out that *A. sinensis* has the most abundant *TPS*-b copies among *Apiaceae* (15 genes, **Supplementary Table S8**). Among these, *AsTPS*8 and *AsTPS*9 form a tandem duplicate pair on Chr3, while *AsTPS*19, *AsTPS*20, and *AsTPS*21 are in a tandem cluster along Chr7. (**Supplementary Figure S3**). Expansion of the *TPS*-b subfamily here might imply potential adaptive response to environments (**Figure 5A**). The *TPS*-c gene, which encodes diterpene synthases involved in the biosynthesis of gibberellin precursors, has only one copy. We evaluated syntenic relationship of *A. sinensis* chromosomes within the *Apiaceae* family using MCScanX. Totally 1,492, 1,168, and 1,022 collinear blocks were identified against *Coriandrum sativum*, *Daucus carota*, and *A. sinensis* itself, respectively (**Figure 5B**).

These *AsTPS* proteins ranged from 204 to 786 amino acids (aa). The molecular weight (MW) ranged from 24.092 to 89.815 kDa, and the predicted isoelectric points (pI) ranged from 4.63 (*A*sTPS22) to 8.69 (*AsT*PS5). Hydrophobicity analysis showed negative GAVY values, indicating that *As*TPS is a hydrophilic protein. Finally, Subcellular localization prediction showed that these *As*TPS proteins were mainly predicted to be localized in the cytoplasm and chloroplast, with a few members predicted in the nucleus, mitochondrion, vacuole, peroxisome, endoplasmic reticulum, or extracellular fluid (**Table 2**).

### 3.5 Functional validation of *AsTPS*s *in vitro*

**Figure 6:**
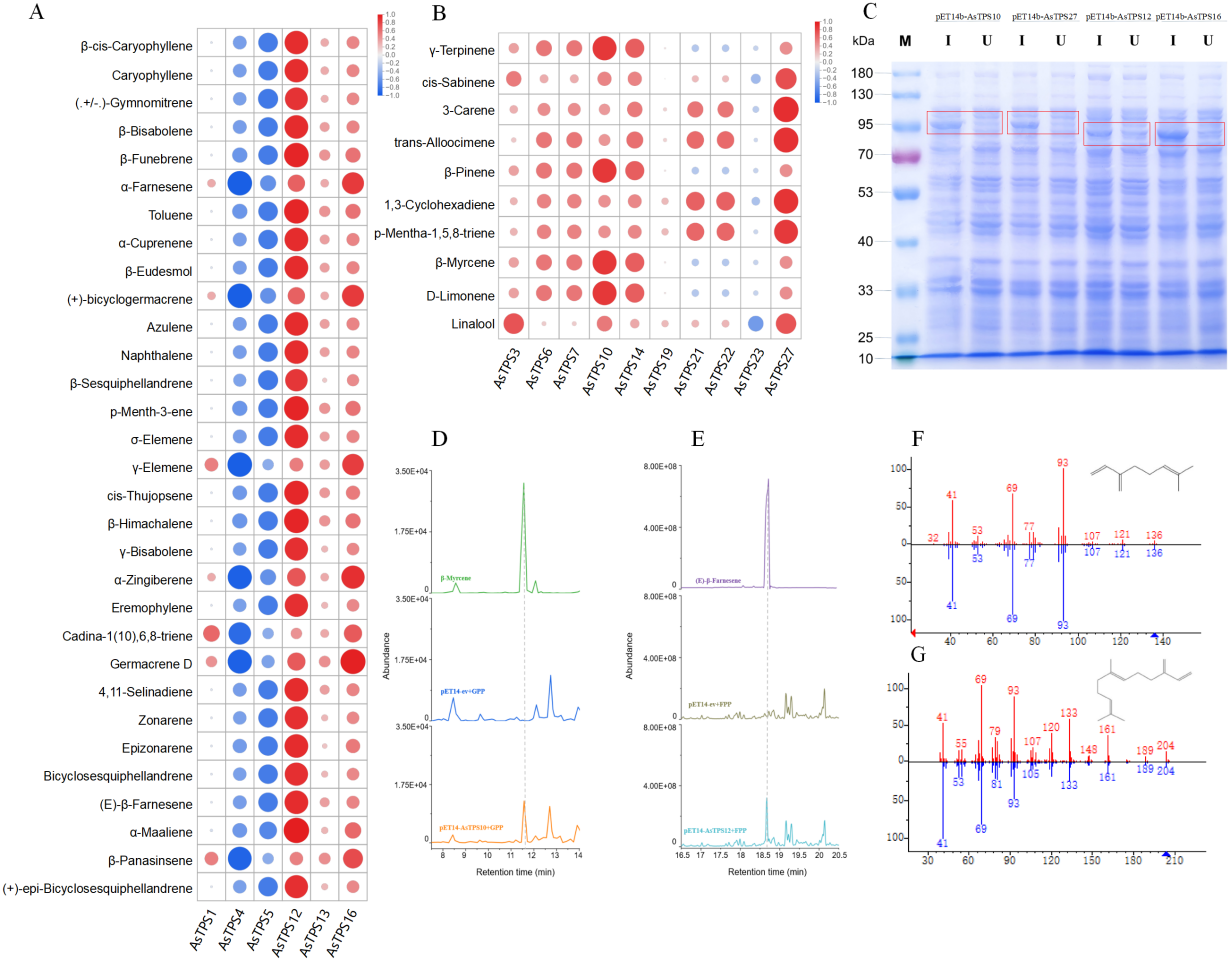
Functional analysis of *As*TPS*s*. **(A)** Correlation bubble heatmap showing the relationship between the gene expression patterns of *AsTPS*-a and sesquiterpene contents in *A. sinensis*. **(B)** Correlation bubble heatmap showing the relationship between the gene expression patterns of *AsTPS*-b and *AsTPS*-g and monoterpene contents in *A. sinensis*. (Circle size represents the correlation coefficient (R) values, and while color denotes the direction of correlation). The diameter of the circles is proportional to the correlation coefficient (*R*), with red and blue denoting positive and negative correlations, respectively. **(C)** Prokaryotic expression analysis of pET14b-*As*TPS10, pET14b-*As*TPS12, pET14b-*As*TPS16, and pET14b-*As*TPS27 in *E. coli* Rosetta (DE3). (I: induced; U: uninduced; M: marker). Red boxes indicate the expected migration regions of the recombinant AsTPS proteins. **(D)** GC-MS profiling of *in vitro* products from *AsTPS*10 enzymatic activity with GPP. (Peak 1 is *β*-*myrcene* with RT 11.65 min). **(E)** GC-MS analysis of *in vitro* products from *As*TPS12 protein activity with FPP (Peak 2 is **(E)**-*β*-*farnesene* with RT 18.76 min). **(F)** *AsTPS*10 enzyme activity product MS spectrum. **(G)** *As*TPS12 enzyme activity product MS spectrum.

To select candidate *AsTPS* genes for terpenoid biosynthesis, we performed a correlation analysis between the expression of the 27 *AsTPS* genes and the content of volatile terpenoids (10 monoterpenes or 31 sesquiterpenes) in *A. sinensis*. This analysis revealed significant correlations (|R|> 0.80 and *p* < 0.05) between five *AsTPS*s (*AsTPS*4, *AsTPS*10, *AsTPS*12, *AsTPS*16, and *AsTPS*27) and volatile terpenoid content (**Figures 6A, B**; **Supplementary Tables S10–S13**). We then selected four highly expressed genes (FPKM≥10), namely *AsTPS*10, *AsTPS*12, *AsTPS*16, and *AsTPS*27, for functional validation. The ORF sequences of these four genes, excluding the signal peptide fragments, were first cloned into pET14b and expressed in *E. coli* Rosetta (DE3). In SDS-PAGE, the molecular weights of the expressed proteins were approximately 98.3 kDa for *As*TPS10, 94 kDa for *AsTPS*12, 92.8 kDa for *As*TPS16, and 98.1 kDa for *As*TPS27, each with a 30.2 kDa His-GFP tag (**Figure 6C**). Then, using GPP or FPP as substrates, enzymatic reactions with the purified fusion proteins were tested. GC-MS was then used to analyze the products.

The purified *As*TPS10 protein, using GPP as the substrate, produced a distinct peak in the GC-MS profile, which was absent in the control reaction expressing empty vector (**Figure 6D**). Comparison with the reference standard confirmed this peak as *β*-*myrcene* based on the retention time and mass spectrum (**Figure 6F**). Similarly, when FPP was used as substrate, the purified protein *As*TPS12 yielded (E)-*β*-*farnesene*, which was confirmed by GC-MS analysis (**Figures 6E, G**). No novel peaks can be detected for *As*TPS*16* and *As*TPS27 protein using FPP or GPP, implying that no activity was detected for these two *As*TPSs protein under the tested *in vitro* conditions.

## 4 Discussion

*A. sinensis* has a long history of medicinal use. Its main component, volatile terpenoids, possesses significant pharmacological activity and economic value. In this study, 10 monoterpenes and 31 sesquiterpenes were identified in three root tissues (periderm, cortex, and stele) through metabolomics. The contents of volatile terpenoids were higher in the root periderm tissues than in its cortex and stele tissues. Moreover, the expression of related genes of monoterpene and sesquiterpene also has corresponding tissue specificity. Previous studies of root-part medicinal plants have focused on the differential expression of genes and metabolites across different tissues (Kerwin et al., 2024; Wang et al., 2024b; Tyagi et al., 2026). For example, ginsenosides are unevenly distributed and accumulated in *Panax plants* (Xu et al., 2017a, 2020a). Ginsenoside levels are higher in berries than in *P. ginseng* roots (Park et al., 2019). Total ginsenoside content is higher in *P. quinquefolius* leaves than in its roots (Yang et al., 2022). These findings align with prior studies.

Terpenoid synthase (TPS) is essential for the biosynthesis of terpenoid compounds and their derivatives. It also contributes significantly to plant growth, development, and stress resistance. With rapid advances in high-throughput sequencing and molecular biology, individual genome screening has been used to obtain *TPS* gene family information in an increasing number of plant species (Chen et al., 2011; Falara et al., 2011; Liu et al., 2024; Xu et al., 2024; Liu et al., 2025; Zhou et al., 2025). Meanwhile, more than 50 species of *TPS* genes have been characterized, and *TPS* functional identification has become a hot topic in the research of plant secondary metabolic pathways (Workman et al., 2018; Cao et al., 2023; Liu et al., 2025).

Conserved domain analysis identified 27 *AsTPS* genes in the *A. sinensis* genome. These results enabled us to investigate their biosynthetic pathways and identify candidate genes. Next, the physicochemical properties, subcellular localization, physical localization, collinearity, and gene family of the *AsTPS* family genes were analyzed. The *AsTPS* family genes of *A. sinensis* can be further subclassified into: *TPS*-a, *TPS*-b, *TPS*-g, *TPS*-c, and *TPS*-e/f. Among them, the *TPS-b* and *TPS-a* have the largest number of members, with 15 and 7, respectively. Similar to the long-leaved mint of the *Dendrobium officinale*, *Freesia*, and *Cymbidium faberi* (Gao et al., 2018; Wang et al., 2021; Cao et al., 2023). There are more *TPS-b* members than *TPS-a* in *A. sinensis*. *TPS-a* members mainly synthesize sesquiterpenes, while *TPS-b* subfamily members primarily produce monoterpenes. This is consistent with the main components of sesquiterpenes and monoterpenes (Wang et al., 2024a; Jin et al., 2025) found in *A. sinensis*. *TPS-a* and *TPS-b* are the two largest subfamilies in *A. sinensis*. However, the functions of *AsTPS* genes related terpenoids and sesquiterpenoids synthesis in *A. sinensis* have not been reported, which warrants further investigation.

Following genomic analysis, the 27 *AsTPS* genes were further subjected to combined metabolomics and transcriptomics co-expression analysis. This analysis identified the *AsTPS*10, *AsTPS*12, *AsTPS*16, and *AsTPS*27 genes as core genes in different root tissues of *A. sinensis*, and all of them were selected for further functional validation. However, no activity was detected for the two proteins *As*TPS16 and *As*TPS27 under the tested *in vitro* conditions. This negative result may be attributed to incorrect substrate selection, misfolded protein, improper truncation, suboptimal reaction conditions, or missing cofactors.

Experimental results show that the *As*TPS10 protein encodes *β*-*myrcene* synthase, while the *As*TPS*12* protein was identified as (E)-*β*-*farnesene* synthase. Both *β*-*myrcene* and (E)-*β*-Farnesene are common volatile terpenoids. The *in vitro* activity of *AsTPS*10 and *As*TPS12 toward GPP confirms their catalytic potential, yet this alone does not clarify their *in vivo* subcellular localization. The precise subcellular localization and physiological functions of *As*TPS10 and *As*TPS12 in *A. sinensis* roots will need further experimental validation, including transient expression of *As*TPS10-GFP or *As*TPS12-GFP fusion proteins and genetic manipulation assays. Furthermore, conducting additional transient silencing experiments or sTable overexpression transformation experiments on *A. sinensis* roots will allow more direct confirmation of the functions of *As*TPS10 and *As*TPS12 protein in the biosynthesis of volatile terpenoids in this plant.

The expression of *β*-*myrcene* synthase is highest in *A. sinensis* the cortex, which corresponds to the highest *β*-*myrcene* content in this tissue. It has antioxidant and stress-resistance activity and shows neuroprotective effects against cerebral ischemia (Shu et al., 2024; Xu et al., 2026). On the other hand, (E)-*β*-*farnesene* synthase exhibits the maximum expression in the *A. sinensis* periderm, and the periderm has the highest (E)-*β*-Farnesene content. (E)-*β*-Farnesene is a plant defense signaling molecule that can effectively reduce the population of pests such as wheat aphids (Khalid et al., 2023). These findings suggest potential targets for future studies on improving monoterpenes and sesquiterpenes biosynthesis in these medicinal plants. Volatile terpenoid pathway engineering in recombinant microbes may enable future commercial production of useful monoterpenes and sesquiterpenes.

## 5 Conclusion

Comprehensive analysis of *A. sinensis* root volatile compounds and transcriptomes identified key genes for terpenoid biosynthesis. Conserved-domain screening identified genes of the final product synthesis enzyme *As*TPS family in the *A. sinensis* genome. An analysis of the physicochemical properties, subcellular localization, physical localization, and collinearity of the *AsTPS* family genes was conducted. Prokaryotic expression vectors were constructed for the four *AsTPS* genes, and enzymatic assays confirmed that the terpene synthases *As*TPS10 and *As*TPS12 catalyze the production of *β*-myrcene and (E)-*β-farnesene* from GPP and FPP, respectively. Together, these findings provide candidate enzymes as a basis for future functional studies in *A. sinensis* and offer a foundation for the future exploration of the biosynthetic pathway and targeted breeding of this medicinal plant.

## Data Availability

The project accession is PRJCA065054. It is publicly available at the Genome Sequence Archive (GSA, NGDC): https://ngdc.cncb.ac.cn/gsa/search?searchTerm=PRJCA065054.
