# Supplementary material for: Genome-wide analysis and functional characterization of AsTPS genes in Chinese angelica (Angelica sinensis)
Source: Front Plant Sci. 2026 Jul 10;17:1872863. doi: 10.3389/fpls.2026.1872863 (PMC13395871; doi:10.3389/fpls.2026.1872863)
Supplement: Supplementary file 1 [file Table1.docx]

**
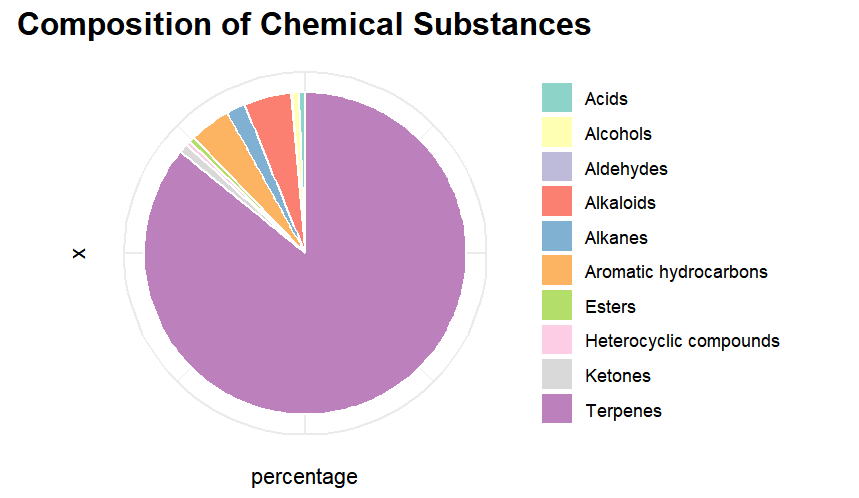
**

**Figure S1.** Volatile compound content in the roots of *A. sinensis.*


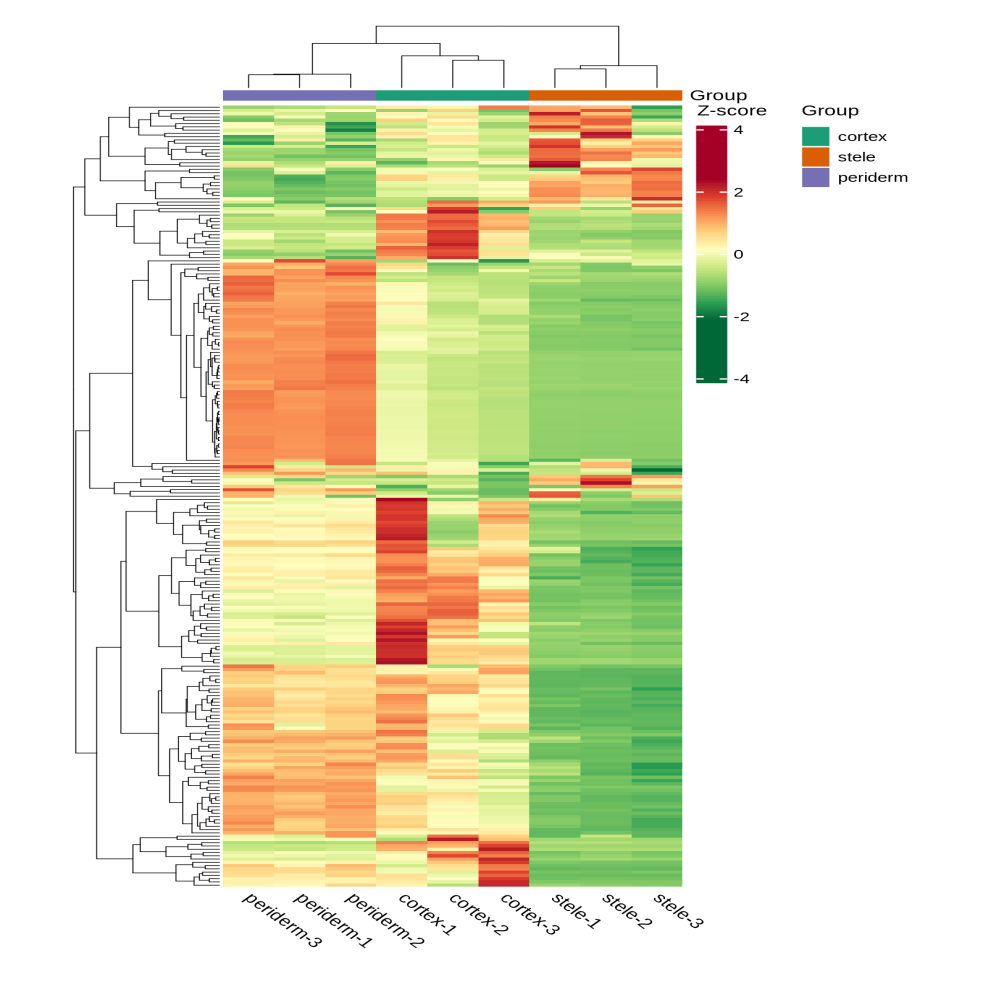


**Figure S2.** Sample clustering graph in different tissues of *A. sinensis* (p: periderm, c: cortex, s: stele)

**
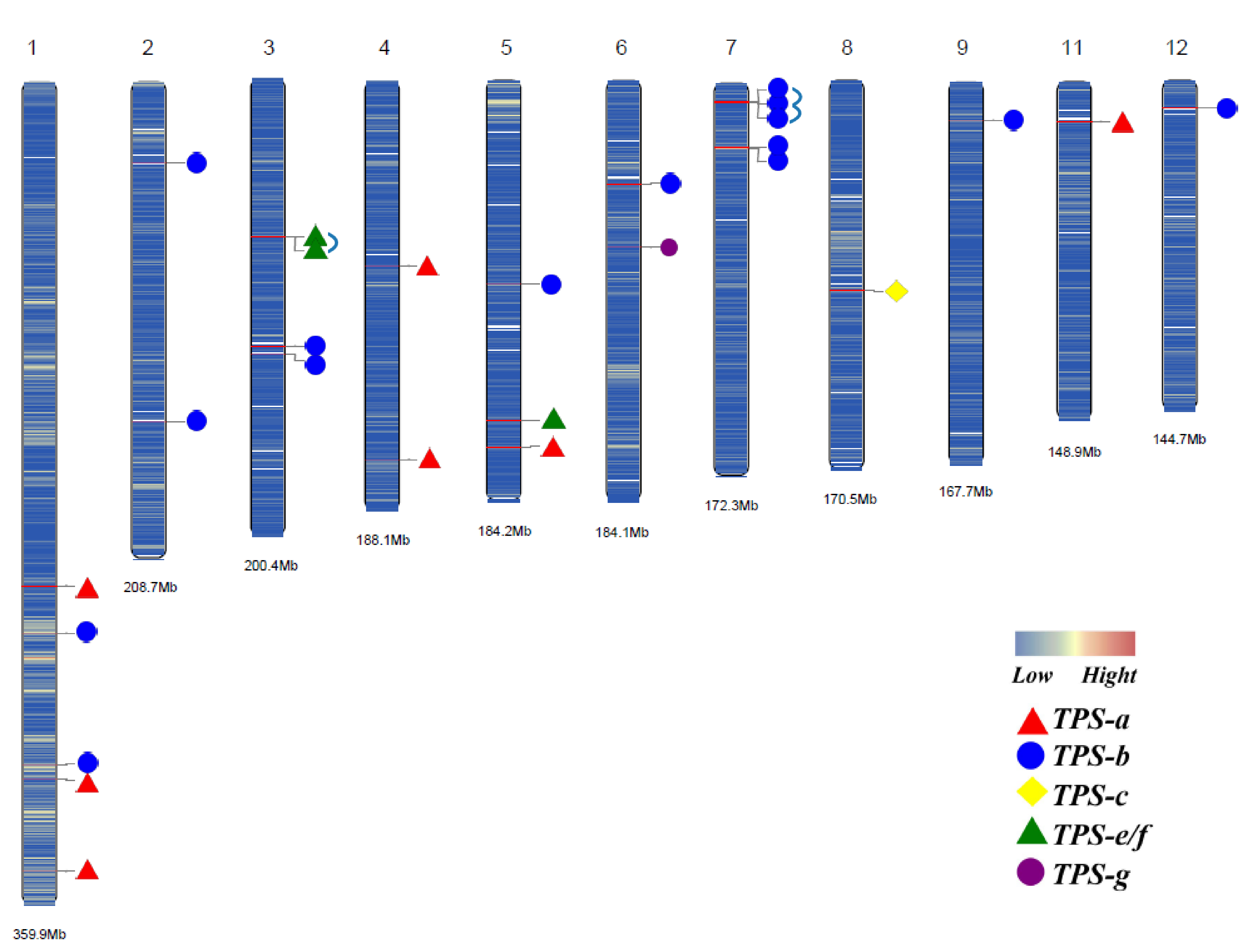
**

**Figure S3.** Distribution of *AsTPS* genes on chromosomes in *A. sinensis.* Tandem duplicated genes are interconnected via blue arcs.

**
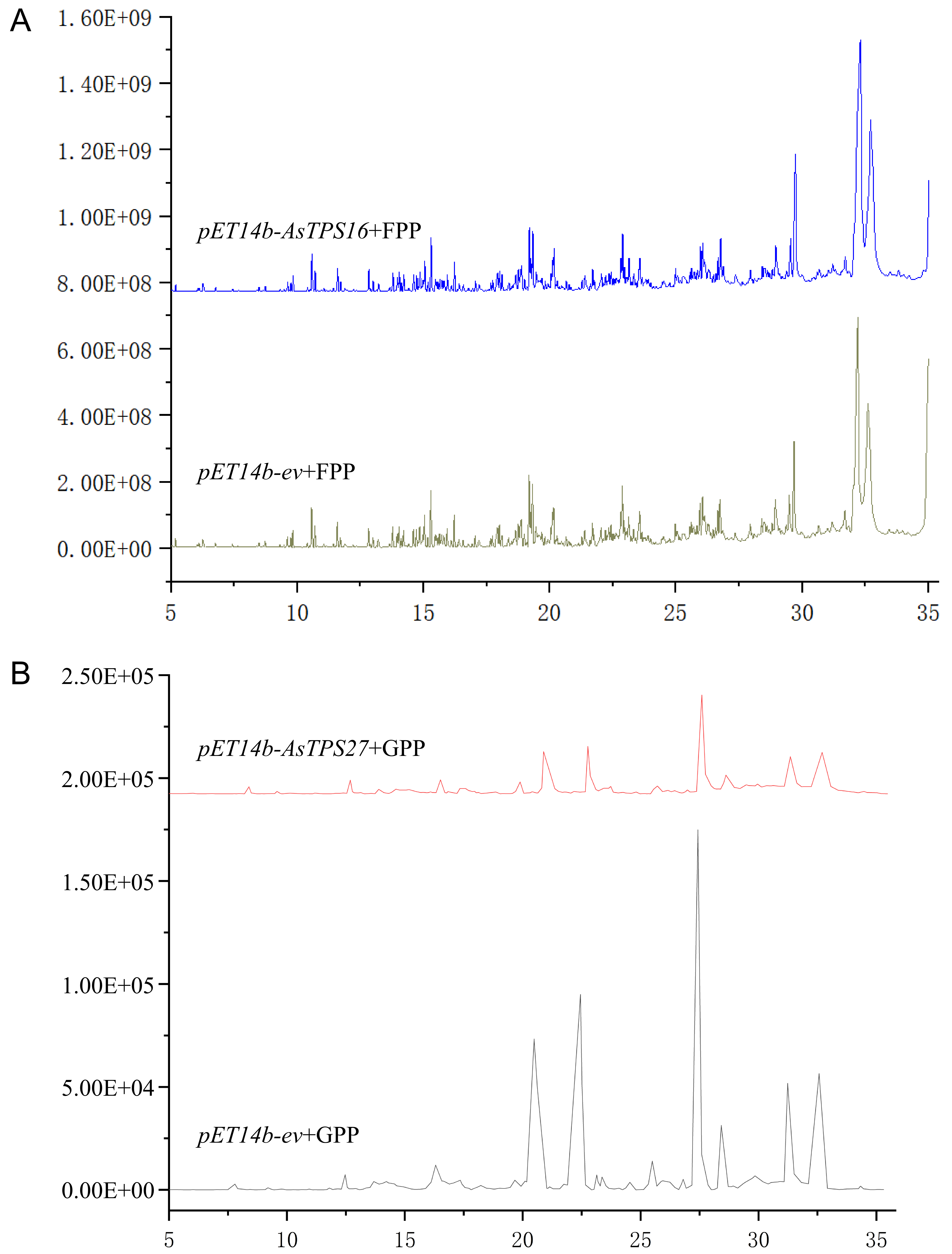
**

**Figure S4.** The GC-MS Analysis of *pET14b-AsTPS16* and *pET14b-AsTPS27.* A, The products generated by the in vitro catalysis of *pET14b-AsTPS16* recombinant protein using FPP as the substrate were identified by gas chromatography-mass spectrometry (GC-MS). B, The products generated by the in vitro catalysis of *pET14b-AsTPS27* recombinant protein using GPP as the substrate were identified by gas chromatography-mass spectrometry (GC-MS).
